# IL-33 Induces a Switch in Intestinal Metabolites Revealing the Tryptophan Pathway as a Target for Inducing Allograft Survival

**DOI:** 10.3390/nu16213655

**Published:** 2024-10-27

**Authors:** Camila Pinto, Tomás Carrasco-Loncharic, Eduardo González-Mienert, Javiera de Solminihac, Felipe Gálvez-Jirón, Federico Cifuentes, Karina Pino-Lagos

**Affiliations:** 1 Facultad de Medicina, Centro de Investigación e Innovación Biomédica, Universidad de los Andes, Santiago 755000, Chile; 2 Escuela de Medicina Veterinaria, Facultad de Agronomía e Ingeniería Forestal, Facultad de Ciencias Biológicas y Facultad de Medicina, Pontificia Universidad Católica de Chile, Santiago 8331150, Chile

**Keywords:** IL-33, T regulatory cells, microbiota, tryptophan, transplantation, tolerance

## Abstract

Background: IL-33, a pleiotropic cytokine, has been associated with a plethora of immune-related processes, both inflammatory and anti-inflammatory. T regulatory (Treg) cells, the main leukocyte population involved in immune tolerance, can be induced by the administration of IL-33, the local microbiota, and its metabolites. Here, we demonstrate that IL-33 drastically induces the production of intestinal metabolites involved on tryptophan (Trp) metabolism. Methods: naïve mice were treated with IL-33 for 4 days and leukocyte populations were analyzed by flow cytometry, and feces were processed for microbiota and intestinal metabolites studies. Using a murine skin transplantation model, the effect of Kynurenic acid (KA) on allograft survival was tested. Results: Under homeostatic conditions, animals treated with IL-33 showed an increment in Treg cell frequencies. Intestinal bacterial abundance analysis indicates that IL-33 provokes dysbiosis, demonstrated by a reduction in *Enterobacteria* and an increment in *Lactobacillus* genera. Furthermore, metabolomics analysis showed a dramatic IL-33 effect on the abundance of intestinal metabolites related to amino acid synthesis pathways, highlighting molecules linked to Trp metabolism, such as kynurenic acid (KA), 5-Hydroxyindoleacetic acid (5-HIAA), and 6-Hydroxynicotinic acid (6-HNA), which was supported by an enhanced expression of *Ido* and *Kat* mRNA in MLN cells, which are two enzymes involved on KA synthesis. Interestingly, animals receiving KA in drinking water and subjected to skin transplantation showed allograft acceptance, which is associated with an increment in Treg cell frequencies. Conclusions: Our study reveals a new property for IL-33 as a modulator of the intestinal microbiota and metabolites, especially those involved with Trp metabolism. In addition, we demonstrate that KA favors Tregs in vivo, positively affecting skin transplantation survival.

## 1. Introduction

IL-33, first recognized as an alarmin because of its release during tissue damage, is a cytokine that belongs to the IL-1 cytokine family. Despite several studies describing its pleiotropic role in different disease models [1], most of the newest reports detail an important function in intestinal homeostasis. It has been demonstrated that IL-33 signaling in T regulatory (Treg) cells is required for their expansion and function [2,3], and mediators such as intestinal microbiota and metabolites have been postulated as playing a role in Treg cell homeostasis in the gut [4,5,6,7]. Hence, Treg cells are one of the most studied cell populations due to their role in self-tolerance and their function in controlling autoimmune diseases, allergic reactions, and transplantation tolerance, among other contributions to health [8]. Although more than one subset of Treg cells have been reported, this study focuses on “conventional” Treg cells, which can be identified by the expression of the transcription factor Forkhead box P3 (FoxP3) [9].

Tryptophan (Trp) is one of the nine essential amino acids present in most protein-based foods that can be metabolized by host cells and gut microbiota. It is mainly absorbed in the small intestine, and in circulation, it is found to be bound to albumin, although a small fraction can be protein-free [10,11]. Trp can be catabolized via three different pathways: the Kynurenine (KYN) pathway, the serotonin (5-Hydroxytryptamine, 5-HT) pathway, and the microbiota pathway. About 95% of Trp is used in the KYN pathway, producing several Kynurenines, such as kynurenic acid (KA), whereas the remaining 5% is shared between the serotonin and microbiota pathways [12].

Cells, including T cells, can consume Trp metabolites through the system L amino acid carrier (SLC) superfamily, which is sensed by G-protein-coupled membrane receptors (GPCRs) expressed in host intestinal cells [13], implying that T cell biology could be modulated by these molecules.

Our group interest is to study immune cells and molecules involved in the generation of immune tolerance. We and others have reported that IL-33 induces transplantation acceptance by favoring the de novo generation of Treg cells [14,15,16]. In human cells, we found that Treg cells cultured and expanded in the presence of IL-33 produced human Treg cells with better suppressive capability [17]. Because of the evidence showing that IL-33 plays an important function in immune tolerance favoring Treg cells under inflammatory conditions, the aim of this report was to investigate whether IL-33 may have an effect under homeostatic conditions. We tested IL-33’s effect on the frequencies of leukocyte populations, microbiota composition, and intestinal metabolites, identifying the Trp pathway as a potential IL-33 target for modulating immunity.

## 2. Materials and Methods

### 2.1. Mice

Six- to eight-week-old wild-type C57BL/6 and FoxP3/GFP (C57BL/6 background) mice were used. All mice were maintained under pathogen-free conditions at a room temperature of 22 °C with a 12 h light/dark cycle and food and water ad libitum (Prolab RMH 3000, LabDiet, Hubbard, OR, USA). For each experiment, the total number of mice (indicated for each experiment in its corresponding figure legend) was randomized to determine the control or experimental groups. Euthanasia was conducted by CO_2_ asphyxiation. In none of the experiments did the animals show signs of pain or distress. This study was carried out after the revision and approval of its experimental protocol (#CEC2021017) in accordance with the recommendations of bioethical committee guidelines from the Faculty of Medicine, Universidad de los Andes, and the Agencia Nacional de Investigación y Desarrollo (ANID).

### 2.2. Skin Transplantation

Tail skin (~1 cm^2^) from C57Bl/6 (syngeneic) or F1 (C57Bl/6 × Balb/c allogeneic) donors was transplanted onto the dorsal area of previously anesthetized C57Bl/6 recipient mice (1 donor mouse provides 6 skin grafts). Anesthesia administration corresponded to a mix of 90 mg/Kg of Ketamine (Troy Laboratories Pty Ltd., Glendenning, Australia) and 10 mg/Kg of Xylazine (Centrovet Ltd., Santiago, Chile) injected in a ratio of 100 mL/30 g body weight via intraperitoneal (i.p) injection. When following this procedure, animals do not experience pain or distress. The survival of skin allografts was evaluated twice per week, and grafts were considered rejected when 80% of the initial graft had disappeared or become necrotic. When indicated, animals received water containing 5 mg/mL of KA (Merck, Rahway, NJ, USA) or water alone. Bottles were refreshed every week.

### 2.3. IL-33 Treatment

Mice were treated with 500 ng of recombinant IL-33 (Peprotech, Cranbury, NJ, USA) from day 0 to day 3 (four injections) via i.p injection. As a control, we administered 100 mL of sterile PBS 1X via i.p injection.

### 2.4. RT-qPCR

Mice were treated as described above. On day 4, mesenteric lymph node (MLN) cells were recovered and subjected to RNA isolation using the RNeasy Kit (Qiagen, Hilden, Germany), following the manufacturer’s instructions. cDNA libraries were prepared with an iScript cDNA synthesis kit (BioRad, Hercules, CA, USA). The mRNA expression levels of *Ido*, *Kat*, and *18s* were measured in a Mx3000P qPCR system (Agilent Technologies, Santa Clara, CA, USA) using SsoFast EvaGreen qPCR Supermix (BioRad) as a fluorescent detector. The nucleotide sequences of the primers used were as followed: *Ido* (Forward 5′ TTT GGG CAC CAA GCC TGA TA 3′, Reverse 5′CCT GAG GTG GTC AGT TCC AC 3′), *Kat* (Forward 5′TGG GAA TAC GTT TGG GGT CG 3′, Reverse 5′AGA TCC AAG GCA CTG CAG AC 3′), and *18s* (Forward 5′ GCC CGA AGC GTT TAC TTT GA 3′, Reverse 5′ TTG CGC CGG TCC AAG AAT TT 3′). For analysis, relative expression was determined using the ΔΔCt method, using 18S as a housekeeping control.

### 2.5. Flow Cytometry

Cell samples were stained with anti-CD4 (clone RM4-5), anti-CD8 (clone 53-6.7), anti-CD19 (clone 6D5), anti-CD11c (clone N418), anti-IA-IE (clone M5/114.15.2), anti-CD11b (M1/70), and anti-CD206 (clone C068C2) (all from BioLegend, San Diego, CA, USA). The anti-Foxp3 antibody (clone FJK-16s) was obtained from eBioscience, San Diego, CA, USA. All antibodies were conjugated with FITC, PE, PE-Cy7, PerCP, PerCP-Cy7, APC, APC-Cy7, or Pacific Blue. All flow cytometry was performed using a BD FACSCanto II cytometer (BD Biosciences, Milpitas, CA, USA), and data were analyzed using FlowJo software (Version 10.10.0) (Tree Star, Girard, OH, USA).

### 2.6. Fecal DNA Isolation and PCR

Fecal material was subjected to the QIAmp Fast DNA Stool isolation kit (Hilden, Germany) following the provider’s instructions. Total DNA was quantified using Nanodrop One C (Thermo Fisher Scientific Inc., Madison, WI, USA), and 10 ng/mL was used to set up the qPCR reaction as published in [18,19,20,21,22]. The primers for the bacterial genes indicated in Table 1 were used. For analysis, relative expression was determined using the ΔΔCt method, using 16S as a housekeeping control. To calculate the relative abundance, we considered the sum of all bacteria genera tested as total microbiota or 100% and then obtained the relative presence for each one.

### 2.7. Fecal Sample Preparation for Ultra-Performance Liquid Chromatography–Mass Spectrometry (UPLC-MS)

Fecal material was collected on day 0 (pre-treatment) and on day 4 (post-treatment with PBS or IL-33) and kept at −80 °C until processing. Animals from the experimental groups were kept in the same cage so the feces could be pooled; thus, one sample corresponds to pooled feces from 4 to 5 mice housed in the same cage. Samples were thawed and added to 500 μL of 80% methanol, and then samples were vortexed for 30 s, followed by sonication for 30 min at 4 °C. All samples were sent to Creative Proteomics (New York, NY, USA) for metabolomic analysis.

### 2.8. UPLC-MS

Separation was performed by Ultimate 3000LC combined with Q Exactive MS (Thermo) and screened with ESI-MS by Creative Proteomics (New York, NY, USA). Briefly, the LC system consisted of an ACQUITY UPLC HSS T3 (100 × 2.1 mm × 1.8 μm) with Ultimate 3000LC. The mobile phase was composed of solvent A (0.05% formic acid water) and solvent B (acetonitrile) with a gradient elution (0–1 min, 95% A, 1–12 min, 95–5% A, 12–13.5 min, 5% A, 13.5–13.6 min, 5–95% A, 13.6–16 min, 95% A). The flow rate of the mobile phase was 0.3 mL·min^−1^. The column temperature was maintained at 40 °C, and the sample manager temperature was set at 4 °C. The mass spectrometry parameters in ESI+ and ESI- mode are listed as follows: ESI+: Heater Temp 300 °C; Sheath Gas Flow Rate, 45 arb; Aux Gas Flow Rate, 15 arb; Sweep Gas Flow Rate, 1 arb; Spray Voltage, 3.0 kV; Capillary Temp, 350 °C; S-Lens RF Level, 30%. ESI-: Heater Temp, 300 °C, Sheath Gas Flow Rate, 45 arb; Aux Gas Flow Rate, 15 arb; Sweep Gas Flow Rate, 1 arb; Spray Voltage, 3.2 kV; Capillary Temp, 350 °C; S-Lens RF Level, 60%. The raw data were acquired and aligned using Compound Discover (3.0, Thermo) based on the m/z value and the retention time of the ion signals. Ions from both ESI- or ESI+ were merged and imported into the SIMCA-P program (version 14.1) for multivariate analysis. A principal component analysis (PCA) was first used as an unsupervised method for data visualization and outlier identification.

### 2.9. Histology

Liver and intestine samples were washed with PBS and then fixed in 10% formalin (HT501128, Sigma Aldrich, St. Louis, MO, USA) for 18 h. Paraffin (Sigma-Aldrich, Merck, Ref. P3683) histo-processing was performed for 24 h. Intestinal sections were oriented transversely and correlative from duodenum to rectum and cut into 3 mm sections using the Accu-cut SRM 200 Rotary Microtome (Sakura Finetek, Torrance, CA, USA). Once the histological slides were obtained, they were left to dry in an oven for 20 min at 60 °C. Sections were stained with hematoxylin (Sigma-Aldrich, H9627) and eosin (Sigma-Aldrich, E4009) and viewed with an Olympus CX31 brightfield trinocular microscope, Olympus Corporation (Tokyo, Japan). Image capturing was performed using a Canon EOS Rebel SL1 and Helicon Remote Software, version 3.9.11 M.

## 3. Statistical Analysis

Statistical analysis was performed using Prism software (Version 10). Mann–Whitney and One-way ANOVA (multiple comparison) tests were used. *p* values < 0.05 were considered statistically significant, and the significance were indicated in the figure legend according to each figure.

## 4. Results

### 4.1. IL-33’s Impacts on Treg Cell Frequencies and Phenotype

IL-33 plays a diverse role in immunity, as demonstrated in various experimental models of disease (including the regulation of intestinal microbiota, immune response to infections, transplantation, among others) [23,24,25].

Based on these antecedents, we sought to investigate whether IL-33 may exert any immunological changes under homeostatic conditions. FoxP3/GFP reporter mice were treated daily with IL-33 or PBS 1X alone. Feces and MLNs were collected for UPLC-MS-based untargeted metabolomics and flow cytometry to identify and quantify intestinal metabolites and cell populations, respectively.

**Figure 1:**
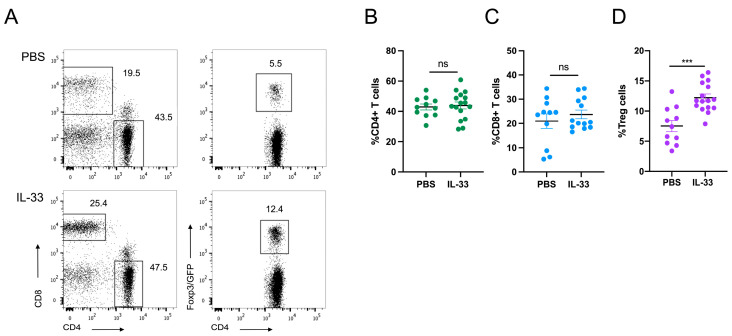
IL-33 increases the frequencies of Tregs with an anti-inflammatory phenotype in the gut. Mice were treated as mentioned above. On day 4, MLNs were removed, and cell suspensions were incubated with antibodies to identify total CD4+ T, CD8+ T cells and CD4+FoxP3^GFP+^ Treg cells. (**A**). Dot plots showing total CD4+ T cells, CD8+ T cells, and CD4+FoxP3+ Treg cells in animals treated with PBS (top plots) and IL-33 (bottom plots). Graphs depicting the frequencies of total CD4+ T cells (**B**), CD8+ T cells (**C**), and CD4+FoxP3^GFP+^ Treg cells (**D**). Each dot represents one animal. Pooled data from 4 independent experiments with n = 2–5 per group. *** *p* < 0.001 according to the Mann–Whitney test; ns, not significant. Bar lines represent mean ± SEM.

No evident macroscopic or microscopic alterations or damage was found in the intestines of the IL-33-treated animals, despite there being few intraepithelial lymphocytes and neutrophils in the jejunum and a mild reduction in goblet cells in the colon, as shown in Figure S1. Because IL-33 was administered in the peritoneum, we decided to focus on the gut, specifically on MLNs. Flow cytometry analysis revealed no changes in the frequencies of total CD4+ T cells (42.87% ± 1.957 for the control group and 43.86% ± 2.279 for the IL-33 group) or total CD8+ T cells (20.99% ± 2.999 for the control group and 23.74% ± 1.779 for the IL-33 group), as shown in Figure 1A–C. Interestingly, we found that IL-33 upregulated the frequencies of Treg cells (7.506% ± 0.929 for the control group versus 12.21% ± 0.596 for the IL-33 group; *p* = 0.0004), as shown in Figure 1D. In addition, we studied potential changes in antigen-presenting cells (APCs). M1- and M2-type macrophages, conventional dendritic cells (cDCs), and B cells were considered, including the expression levels of MHC-II in cDCs and B cells. No differences in the frequencies of M1 (67.01% ± 2.348 for the control group and 62.04% ± 2.092 for the IL-33 group) and M2 (5.56% ± 1.310 for the control group and 4.705% ± 0.829 for the IL-33 group) were observed, as shown in Figure S2A–D. Similarly, no differences in the frequencies of cDCs (7.473% ± 0.989 for the control group and 7.765% ± 0.835 for the IL-33 group), as shown in Figure S2E–H, or B cells (10.57% ± 1.196 for the control group and 14.88% ± 2.173 for the IL-33 group), as shown in Figure S1I–K, were found. The levels of MHC-II expression in cDCs (MFI of 19,010 ± 1697 for the control group and 18,483 ± 1779 for the IL-33 group) and B cells (MFI of 24,889 ± 1763 for the control group and 21,969 ± 1534 for the IL-33 group) remained unchanged. Overall, our results indicate that IL-33 increases Treg cell frequencies in MLNs under homeostatic conditions with no overt effect on APC populations.

### 4.2. IL-33 Modulates Intestinal Microbiota and Metabolites

**Figure 2:**
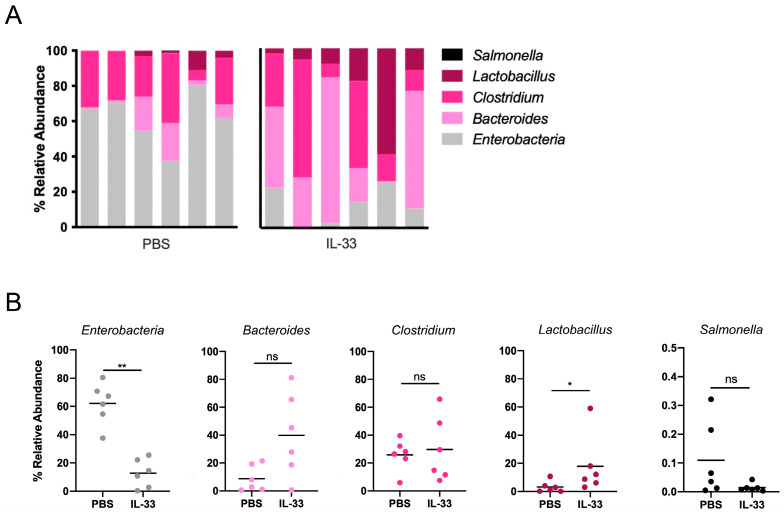
IL-33 induces intestinal dysbiosis. Animals were treated as mentioned above. Pooled fecal material per cage/treatment was subjected to DNA isolation. Primers for the mentioned genera were used considering Eubacteria as a housekeeping control (see Table 1 for primer sequences). (**A**). Percentage relative abundance for the mentioned bacterial genus on animals treated with PBS (left bar graph) or IL-33 (right bar graph). Each bar represents one cage with 4–5 animals. (**B**). Graphs displaying the values obtained per condition for each genus. * *p* < 0.05 and ** *p* < 0.01 according to the Mann–Whitney test; ns, not significant. Bar lines represent mean ± SEM.

Several studies have demonstrated that not only intestinal microbiota but also microbiota–host cell interactions (and the metabolites released during this crosstalk) are pivotal for the maintenance of gut homeostasis [26]. In fact, several reports have described the interactions between intestinal microbiota and Treg cells, along with metabolites such as short-chain fatty acids (SCFAs), among others, demonstrating a dependence between microorganisms and the differentiation and function of Treg cells, which has important consequences on the inflammatory status of the intestinal microenvironment [27,28,29]. Due to the changes found in Treg cells, we decided to evaluate the composition of bacterial microbiota, focusing on bacteria genera involved in pro- and anti-inflammatory events [30]. Fecal DNA from the control and IL-33-treated animals revealed high abundances of *Enterobacteria* (62.07% ± 6.060) and *Clostridium* (25.86% ± 4.616) bacteria, with a low presence of *Bacteroides* (8.823% ± 3.811), *Lactobacillus* (3.138% ± 1.646), and *Salmonella* (0.109% ± 0.053) in the control animals, as shown in Figure 2A, specifically the graph on the left, and Figure 2B. Interestingly, the animals treated with IL-33 showed a reduction in the abundance of *Enterobacteria* (12.68% ± 4.151, *p* = 0.0022) and *Salmonella* (0.014% ± 0.005) but an increment in *Bacteroides* (39.84% ± 12.28) and *Lactobacillus* (17.85% ± 8.506, *p* = 0.026), as shown in Figure 2A, specifically the graph on the right, and Figure 2B. No changes in the abundance of *Clostridium* were detected (29.61% ± 9.498). These data indicate that IL-33 induces intestinal dysbiosis under homeostatic conditions.

**Figure 3:**
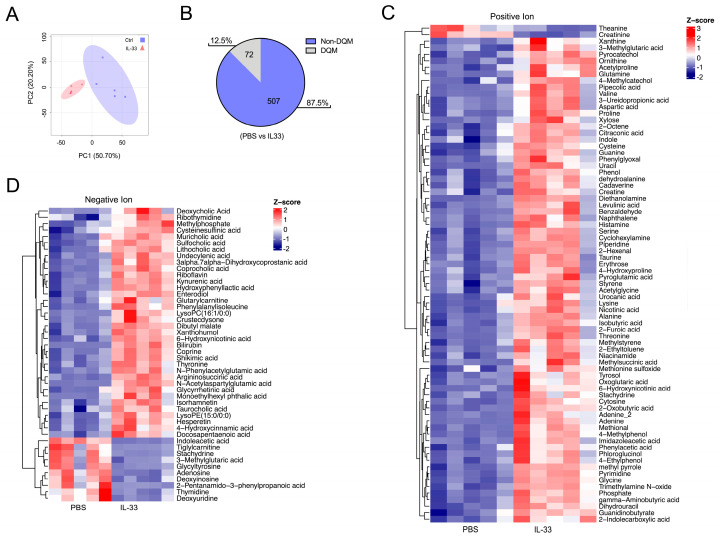
IL-33 impacts the abundance of intestinal metabolites. Fecal material was obtained, as shown in Figure 2. (**A**). PCA plot depicting the distribution of metabolites identified in the control (blue) and IL-33-treated mice (red). (**B**). Pie plot displaying the number and percentages of total and different quantitative metabolites (DQMs). Heatmaps showing molecules detected in positive (**C**) and negative (**D**) ion modes in the control and IL-33 groups. Pooled data of five independent experiments with n = 5 animals per group.

To complement these results, feces from the control and IL-33-treated animals were subjected to LC-MS-based untargeted metabolomics. The principal component analysis (PCA) of 579 identified metabolites from five independent experiments showed differences between the control and IL-33-treated groups, resulting in 72 different quantitative metabolites (DQMs), as shown in Figure 3A,B. Heatmap visualization of the DQMs between the control and IL-33-treated groups reveals two major clusters in positive and negative ion mode, as shown in Figure 3C,D, respectively.

**Figure 4:**
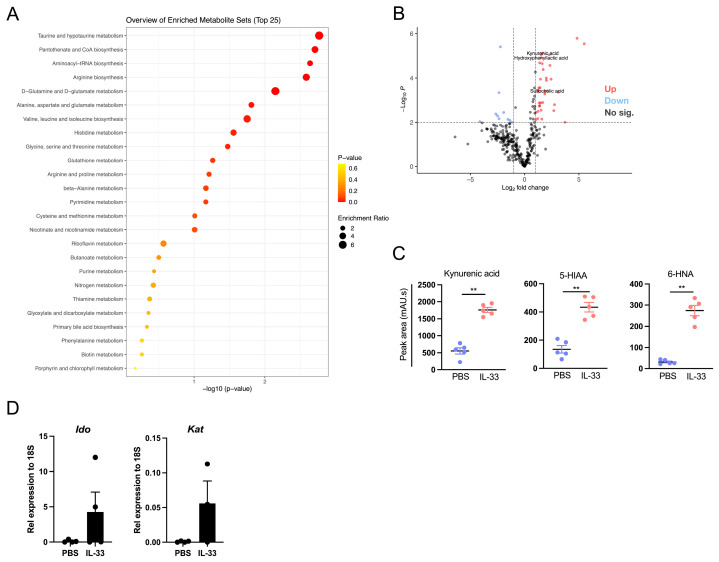
IL-33 induces the production of intestinal molecules linked to Trp metabolism. Fecal material was obtained as shown in Figure 2. (**A**). KEGG analysis of DQMs between PBS and IL-33-treated cells. The DQMs were identified based on a fold change between the IL-33 and PBS groups of more than 2.0 or less than 0.5. (**B**). Volcano plot depicting DQMs linked to different Trp metabolites between PBS- and IL-33-treated mice. (**C**). Abundance of DQMs of selected molecules. (**D**). qPCR for *Ido* and *Kat* on MLN cells from control (PBS) and IL-33 groups. Metabolomic studies correspond to pooled data of five independent experiments with n = 5 animals per group. qPCR data correspond to 2 independent experiments with n = 2 animals per group. ** *p* < 0.01 according to the Mann–Whitney test. Bar lines represent mean ± SEM.

Kyoto Encyclopedia of Genes and Genomes (KEGG) enrichment analysis revealed that the metabolic changes in the IL-33-treated animals were mainly involved in amino acid metabolism (Taurine, Glutamine/Glutamate, Valine/Leucine/Isoleucine), Arginine biosynthesis, and Pantothenate/CoA biosynthesis, as shown in Figure 4A.

Using volcano plots showing the DQMs between the control and IL-33-treated groups, we identified at least three highly enriched signature molecules involved in tryptophan metabolism, a key amino acid linked to immune regulation [12]—Kynurenic acid (KA), 5-Hydroxyindoleacetic acid (HIAA), and 6-Hydroxynicotinic acid (6-HNA)—as shown in Figure 4B,C. Tryptophan (Trp) is an essential amino acid required for protein synthesis and serves as a precursor of metabolites involved in several functions, including immunity. Trp can take three different metabolic pathways—Kynurenic acid (KA), in which the enzymes indolamine 2,3-dioxygenase (IDO) and Kynurenine aminotransferase (KAT) are involved; serotonin (5-HT); and gut microbiota generation of indole derivatives—but in mammalian cells, the KA pathway is the main route [12]. Since our data show that IL-33 triggers the upregulation of Trp-related metabolites, we evaluated the expression of the *Ido* and *Kat* genes in the MLNs of the PBS- and IL-33-treated animals, finding a tendency of ~5-fold upregulation in both genes for the animals receiving IL-33 injections, as shown in Figure 4D.

### 4.3. Kynurenic Acid, a Trp Metabolite, Induces Treg Cells and Favors Allograft Survival in Transplanted Animals

**Figure 5:**
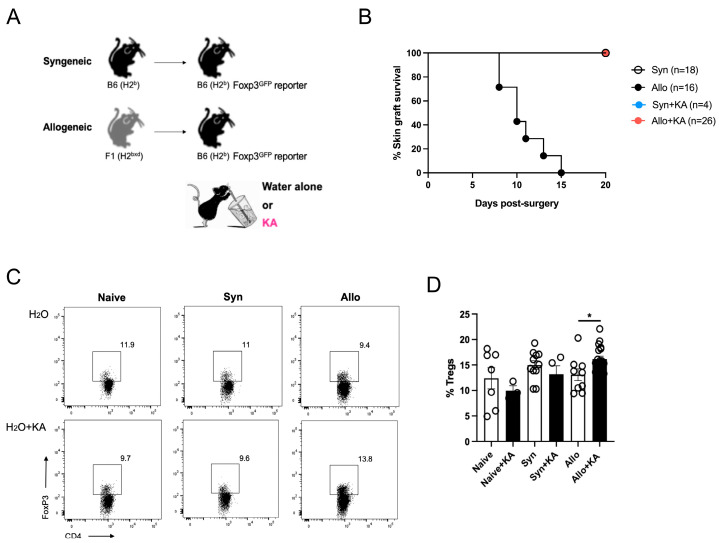
Kynurenic acid induces skin transplant acceptance. (**A**). C57Bl/6-recipient animals received a syngeneic (C57BL/6 skin) or allogeneic (C57BL/6 × Balb/c F1 skin) transplant onto the dorsal area. Control groups received normal water, and experimental groups received water supplemented with KA (5 mg/L). The survival of skin allografts was evaluated twice per week, and grafts were considered rejected when 80% of the initial graft had disappeared or become necrotic. On day 20, skin graft-draining lymph nodes (dLNs) were removed for flow cytometry analysis. (**B**). Skin transplant survival. (**C**). Representative dot plots depicting the frequencies of Treg cells, which were identified by intracellular staining. (**D**). Bar plots showing cumulative Treg cell data. Each dot corresponds to a mouse. Pooled data of four independent experiments. * *p* < 0.05 according to two-way ANOVA (Tukey’s test). Bar lines represent mean ± SEM.

Based on the finding that IL-33 administration results in an increment in KA, a Trp metabolite that stimulates a Treg cell/anti-inflammatory gene signature in mice [31,32], we decided to evaluate the potential immunoregulatory function of KA in a murine skin transplantation model. On day 0, the C57BL/6 mice received syngeneic or allogeneic skin grafts and were offered normal water or water supplemented with 5 mg/L of KA, as shown in Figure 5A. It is important to note that water consumption was not affected by the inclusion of KA and that no signs of liver damage (cell infiltration, necrosis, or inflammation) were observed in the experimental animals, as shown in Figure S3A,B. On day 20 post-surgery, skin-draining lymph nodes (axillary and brachial, dLNs) were removed, and Treg cell frequencies were determined by flow cytometry. As shown in Figure 5B, we found that KA prevents skin graft rejection. Flow cytometry analysis of dLNs showed that the mice transplanted with an allograft and receiving KA in their water had more Treg cells than the control animals (16.17% ± 0.552 versus 13.09% ± 1.166, respectively), as shown in Figure 5C,D, revealing a new immune regulatory function for KA.

## 5. Discussion

Several groups have studied the role of IL-33 in the induction and/or maintenance of immune tolerance. Besides revealing the effect of IL-33 in transplantation immunology, other investigations have shown that IL-33 signals directly to gut-associated Treg cells, and this is required for Treg cell generation and function [2], demonstrating the potential benefits of IL-33 activity in gut-/intestinal inflammatory-related pathologies. In this study, we report that the injection of IL-33 enhanced Treg cell frequencies under homeostatic conditions without affecting the populations of total CD4+ T and CD8+ T cells or other leukocyte subsets, as shown Figure 1. Although this finding is in line with previous reports, it is limited since we did not determine whether these Treg cells are differentiated from CD4+FoxP3− T cells (de novo Treg cells) or expand from the thymus-derived or natural Treg cell pool. In addition, other questions remain open, such as the phenotype and function of the Treg cells post-IL-33 treatment, among others.

Along with this effect, we also found that the populations of gut bacteria become influenced by the presence of IL-33, as shown in Figure 2. Specifically, the animals receiving IL-33 displayed a robust switch in the abundance of, at least, *Enterobacteria* (reduction) and *Bacteroides*, in addition to *Lactobacillus* (increment), uncovering a potential new role for IL-33 as an inducer of microbiota with regulatory or tolerogenic characteristics. In fact, *Enterobacteria* species have been associated with intestinal inflammation [33,34], whereas both *Bacteroides* and *Lactobacillus* may play a beneficial function in intestinal homeostasis [35,36,37,38]. Furthermore, some species of *Bacteroides* and *Lactobacillus* genera can also catabolize Trp to produce molecules that act as AhR agonists [39]. Because some studies have also demonstrated that bacteria express molecules that sense eukaryotic cytokines [40,41], there is a possibility that IL-33 may act directly on them, affecting microbiota composition, or on host (eukaryotic) cells or both, but this point needs further research. Future experiments considering massive 16S rRNA sequencing may prove more informative, as the analysis reported here is limited to the mentioned genera. This strategy may reveal IL-33-sensitive species, which may serve as therapeutic targets. Also, we carried out untargeted metabolomic analysis of fecal material from the control and IL-33-treated mice, observing a striking IL-33 effect on the abundance of several molecules, as shown in Figure 3 and Figure 4. The KEGG pathway analysis indicates that some of the molecules positively regulated by IL-33 are involved in amino acid metabolic pathways, especially Trp, a key essential amino acid that plays a pivotal role in immunity [12]. For instance, KA, 5-HIAA, and 6-HNA [5,42,43,44] were incremented by four-, two-, and six-fold, respectively, suggesting that IL-33 signaling may trigger Trp metabolism. Interestingly, we were able to detect elevated copies of *Ido* and *Kat* genes in the MLN cells from the IL-33-treated animals, suggesting that IL-33 may affect Trp levels and increment the amount of KA through the upregulation of *Ido* and *Kat* gene expression, as shown in Figure 4D. Another possibility is that IL-33 may enhance IDO and KAT enzymatic activity, resulting in elevated KA, but this hypothesis was not tested in this study. Additional experiments sorting for specific intestinal leukocyte populations and testing for Trp metabolic activity are pending. We are currently developing a method to measure KA from different sources as a way of tracking its synthesis/release under inflammatory conditions.

Lastly, we evaluated the potential role of KA, one of the metabolites enriched in the gut of the IL-33-treated animals, in an in vivo model of skin transplantation. Mice receiving skin allografts and KA in their water showed increased transplant survival, which was associated with an upregulation of the frequencies of FoxP3+ Tregs, as shown in Figure 5. Limitations in this part of the study include the control of KA administration (drinking water instead of injections), as the consumption per animal may have not been equal, and the more comprehensive characterization of Treg cells found in the allogenic group treated with KA.

Since KA acts as a ligand for AhR, it is possible that KA may signal to CD4+ T cells to favor Treg cell differentiation through the Kynurenine pathway [45]. Alternatively, KA may be sensed by GPR35 and regulate anti-inflammatory cytokines, which could favor the presence of Treg cells [32]. Whether there is crosstalk between IL-33 signaling, Trp metabolism, and the outcome of CD4+ T cell differentiation is not fully revealed in this study, but our current investigation is dedicated to understanding this potential mechanism of immune regulation. Nonetheless, this report indicates that IL-33, besides inducing Treg cells, correlates with an immune regulatory signature from intestinal microbiota and metabolites, highlighting the role of KA as a potential molecule to favor transplant tolerance.

## 6. Conclusions

Our study demonstrates that IL-33 causes immune-related changes under homeostatic conditions. It also indicates that an inflammatory stimulus is not required for IL-33 to induce the upregulation of Treg cells or intestinal dysbiosis. In addition, IL-33 stimulates the Trp metabolism, which could be manipulated to prevent or treat pathological conditions.

## Figures and Tables

**Table 1:** Quantitative real-time PCR primer sequences for microbiota abundance analysis.

Target	Sequence (5′–3′)
*Salmonella*	F: TGTTGTGGTTAATAACCGCA
	R: GACTACCAGGGTATCTAATCC
*Lactobacillus*	F: AGCAGTAGGGAATCTTCCA
	R: CACCGCTACACATGGAG
*Clostridium*	F: ACTCCTACGGGAGGCAGC
	R: GCTTCTTAGTCAGGTACCGTCAT
*Bacteroides*	F: GGTTCTGAGAGGAGGTCCC
	R: GCTGCCTCCCGTAGGAGT
*Enterobacteria*	F: GTGCCAGCMGCCGCGGTAA
	R: GCCTCAAGGGCACAACCTCCAAG
*16S*	F: AGAGTTTGATCMTGGCTCAG
	R: AAGGAGGTGWTCCARCC

## Data Availability

The original contributions presented in the study are included in the article/Supplementary Material, further inquiries can be directed to the corresponding author.

## Supplementary Materials

The following supporting information can be downloaded at: https://www.mdpi.com/article/10.3390/nu16213655/s1, Figure S1. Effect of IL-33 on antigen-presenting cells. Mice were treated as mentioned in Figure 1; Figure S2. Intestinal histological analysis of IL-33-treated mice; Figure S3. Effect of KA on skin transplant animals.

## References

[1] Carrasco T.G., Morales R.A., Pérez F., Terraza C., Yáñez L., Campos-Mora M., Pino-Lagos K. (2015). Alarmin’ Immunologists: IL-33 as a Putative Target for Modulating T Cell-Dependent Responses. Front. Immunol..

[2] Schiering C., Krausgruber T., Chomka A., Fröhlich A., Adelmann K., Wohlfert E.A., Pott J., Griseri T., Bollrath J., Hegazy A.N. (2014). The Alarmin IL-33 Promotes Regulatory T-Cell Function in the Intestine. Nature.

[3] Duan L., Chen J., Zhang H., Yang H., Zhu P., Xiong A., Xia Q., Zheng F., Tan Z., Gong F. (2012). Interleukin-33 Ameliorates Experimental Colitis through Promoting Th2/Foxp3+ Regulatory T-Cell Responses in Mice. Mol. Med..

[4] Hand T., Belkaid Y. (2010). Microbial Control of Regulatory and Effector T Cell Responses in the Gut. Curr. Opin. Immunol..

[5] Laursen M.F., Sakanaka M., von Burg N., Mörbe U., Andersen D., Moll J.M., Pekmez C.T., Rivollier A., Michaelsen K.F., Mølgaard C. (2021). Bifidobacterium Species Associated with Breastfeeding Produce Aromatic Lactic Acids in the Infant Gut. Nat. Microbiol..

[6] Campbell C., McKenney P.T., Konstantinovsky D., Isaeva O.I., Schizas M., Verter J., Mai C., Jin W.B., Guo C.J., Violante S. (2020). Bacterial Metabolism of Bile Acids Promotes Generation of Peripheral Regulatory T Cells. Nature.

[7] Hang S., Paik D., Yao L., Kim E., Jamma T., Lu J., Ha S., Nelson B.N., Kelly S.P., Wu L. (2019). Bile Acid Metabolites Control TH17 and Treg Cell Differentiation. Nature.

[8] Ohkura N., Kitagawa Y., Sakaguchi S. (2013). Development and Maintenance of Regulatory T Cells. Immunity.

[9] Zhao H., Liao X., Kang Y. (2017). Tregs: Where We Are and What Comes Next?. Front. Immunol..

[10] Su X., Gao Y., Yang R. (2022). Gut Microbiota-Derived Tryptophan Metabolites Maintain Gut and Systemic Homeostasis. Cells.

[11] Roth W., Zadeh K., Vekariya R., Ge Y., Mohamadzadeh M. (2021). Tryptophan Metabolism and Gut-Brain Homeostasis. Int. J. Mol. Sci..

[12] Modoux M., Rolhion N., Mani S., Sokol H. (2021). Tryptophan Metabolism as a Pharmacological Target. Trends Pharmacol. Sci..

[13] Klaessens S., Stroobant V., De Plaen E., Van den Eynde B.J. (2022). Systemic Tryptophan Homeostasis. Front. Mol. Biosci..

[14] Turnquist H.R., Zhao Z., Rosborough B.R., Liu Q., Castellaneta A., Isse K., Wang Z., Lang M., Beer Stolz D., Zheng X.X. (2011). IL-33 Expands Suppressive CD11b+ Gr-1int and Regulatory T Cells, Including ST2L+ Foxp3+ Cells, and Mediates Regulatory T Cell-Dependent Promotion of Cardiac Allograft Survival. J. Immunol..

[15] Gajardo T., Morales R.A., Campos-Mora M., Campos-Acuña J., Pino-Lagos K. (2015). Exogenous Interleukin-33 Targets Myeloid-Derived Suppressor Cells and Generates Periphery-Induced Foxp3+ Regulatory T Cells in Skin-Transplanted Mice. Immunology.

[16] Kawai K., Uchiyama M., Hester J., Issa F. (2021). IL-33 Drives the Production of Mouse Regulatory T Cells with Enhanced in Vivo Suppressive Activity in Skin Transplantation. Am. J. Transplant..

[17] Gajardo T., Campos-Mora M., Pino-Lagos K. (2017). IL-33 Improves the Suppressive Capacity of Human Regulatory T Cells. Trends Transplant..

[18] Resendiz-Nava C.N., Silva-Rojas H.V., Rebollar-Alviter A., Rivera-Pastrana D.M., Mercado-Silva E.M., Nava G.M. (2022). A Comprehensive Evaluation of Enterobacteriaceae Primer Sets for Analysis of Host-Associated Microbiota. Pathogens.

[19] Engevik M.A., Aihara E., Montrose M.H., Shull G.E., Hassett D.J., Worrell R.T. (2013). Loss of NHE3 Alters Gut Microbiota Composition and Influences Bacteroides Thetaiotaomicron Growth. Am. J. Physiol. Gastrointest. Liver Physiol..

[20] Atif S.M., Winter S.E., Winter M.G., McSorley S.J., Bäumler A.J. (2014). Salmonella Enterica Serovar Typhi Impairs CD4 T Cell Responses by Reducing Antigen Availability. Infect. Immun..

[21] Chan K.G., Atkinson S., Mathee K., Sam C.K., Chhabra S.R., Cmara M., Koh C.L., Williams P. (2011). Characterization of N-Acylhomoserine Lactone-Degrading Bacteria Associated with the *Zingiber Officinale* (Ginger) Rhizosphere: Co-Existence of Quorum Quenching and Quorum Sensing in Acinetobacter and Burkholderia. BMC Microbiol..

[22] Qing X., Zeng D., Wang H., Ni X., Liu L., Lai J., Khalique A., Pan K., Jing B. (2017). Preventing Subclinical Necrotic Enteritis through *Lactobacillus Johnsonii* BS15 by Ameliorating Lipid Metabolism and Intestinal Microflora in Broiler Chickens. AMB Express.

[23] Malik A., Sharma D., Zhu Q., Karki R., Guy C.S., Vogel P., Kanneganti T.D. (2016). IL-33 Regulates the IgA-Microbiota Axis to Restrain IL-1α–Dependent Colitis and Tumorigenesis. J. Clin. Investig..

[24] Xiao Y., Huang X., Zhao Y., Chen F., Sun M., Yang W., Chen L., Yao S., Peniche A., Dann S.M. (2019). Interleukin-33 Promotes REG3γ Expression in Intestinal Epithelial Cells and Regulates Gut Microbiota. Cell. Mol. Gastroenterol. Hepatol..

[25] Röwekamp I., Maschirow L., Rabes A., Vernengo F.F., Hamann L., Heinz G.A., Mashreghi M.F., Caesar S., Milek M., Fonseca A.C.F. (2024). IL-33 Controls IL-22-Dependent Antibacterial Defense by Modulating the Microbiota. Proc. Natl. Acad. Sci. USA.

[26] Yoon J.H., Do J.S., Velankanni P., Lee C.G., Kwon H.K. (2023). Gut Microbial Metabolites on Host Immune Responses in Health and Disease. Immune Netw..

[27] Atarashi K., Tanoue T., Shima T., Imaoka A., Kuwahara T., Momose Y., Cheng G., Yamasaki S., Saito T., Ohba Y. (2011). Induction of Colonic Regulatory T Cells by Indigenous Clostridium Species. Science.

[28] Geuking M.B., Cahenzli J., Lawson M.A.E., Ng D.C.K., Slack E., Hapfelmeier S., McCoy K.D., Macpherson A.J. (2011). Intestinal Bacterial Colonization Induces Mutualistic Regulatory T Cell Responses. Immunity.

[29] Smith P.M., Howitt M.R., Panikov N., Michaud M., Gallini C.A., Bohlooly Y.M., Glickman J.N., Garrett W.S. (2013). The Microbial Metabolites, Short-Chain Fatty Acids, Regulate Colonic T Reg Cell Homeostasis. Science.

[30] Cristofori F., Dargenio V.N., Dargenio C., Miniello V.L., Barone M., Francavilla R. (2021). Anti-Inflammatory and Immunomodulatory Effects of Probiotics in Gut Inflammation: A Door to the Body. Front. Immunol..

[31] Wang J., Simonavicius N., Wu X., Swaminath G., Reagan J., Tian H., Ling L. (2006). Kynurenic Acid as a Ligand for Orphan G Protein-Coupled Receptor GPR35. J. Biol. Chem..

[32] Agudelo L.Z., Ferreira D.M.S., Cervenka I., Bryzgalova G., Dadvar S., Jannig P.R., Pettersson-Klein A.T., Lakshmikanth T., Sustarsic E.G., Porsmyr-Palmertz M. (2018). Kynurenic Acid and Gpr35 Regulate Adipose Tissue Energy Homeostasis and Inflammation. Cell Metab..

[33] Garrett W.S., Gallini C.A., Yatsunenko T., Michaud M., Dubois A., Delaney M.L., Punit S., Karlsson M., Bry L., Glickman J.N. (2010). Enterobacteriaceae Act in Concert with the Gut Microbiota to Induce Spontaneous and Maternally Transmitted Colitis. Cell Host Microbe.

[34] Baldelli V., Scaldaferri F., Putignani L., Del Chierico F. (2021). The Role of Enterobacteriaceae in Gut Microbiota Dysbiosis in Inflammatory Bowel Diseases. Microorganisms.

[35] Wang K., Dong H., Qi Y., Pei Z., Yi S., Yang X., Zhao Y., Meng F., Yu S., Zhou T. (2017). *Lactobacillus casei* Regulates Differentiation of Th17/Treg Cells to Reduce Intestinal Inflammation in Mice. Can. J. Vet. Res..

[36] Telesford K.M., Yan W., Ochoa-Reparaz J., Pant A., Kircher C., Christy M.A., Begum-Haque S., Kasper D.L., Kasper L.H. (2015). A Commensal Symbiotic Factor Derived from Bacteroides Fragilis Promotes Human CD39+Foxp3+ T Cells and Treg Function. Gut Microbes.

[37] Fu L., Peng J., Zhao S., Zhang Y., Su X., Wang Y. (2017). Lactic Acid Bacteria-Specific Induction of CD4+Foxp3+T Cells Ameliorates Shrimp Tropomyosin-Induced Allergic Response in Mice via Suppression of MTOR Signaling. Sci. Rep..

[38] Ding Y.-H., Qian L.-Y., Pang J., Lin J.-Y., Xu Q., Wang L.-H., Huang D.-S., Zou H., Ding Y.-H., Qian L.-Y. (2017). The Regulation of Immune Cells by Lactobacilli: A Potential Therapeutic Target for Anti-Atherosclerosis Therapy. Oncotarget.

[39] Roager H.M., Licht T.R. (2018). Microbial Tryptophan Catabolites in Health and Disease. Nat. Commun..

[40] Mahdavi J., Royer P.J., Sjölinder H.S., Azimi S., Self T., Stoof J., Wheldon L.M., Brännström K., Wilson R., Moreton J. (2013). Pro-Inflammatory Cytokines Can Act as Intracellular Modulators of Commensal Bacterial Virulence. Open Biol..

[41] O’Mahony L., O’Callaghan L., McCarthy J., Shilling D., Scully P., Sibartie S., Kavanagh E., Kirwan W.O., Redmond H.P., Collins J.K. (2006). Differential Cytokine Response from Dendritic Cells to Commensal and Pathogenic Bacteria in Different Lymphoid Compartments in Humans. Am. J. Physiol. Gastrointest. Liver Physiol..

[42] Wang J., Zhu N., Su X., Gao Y., Yang R. (2023). Gut-Microbiota-Derived Metabolites Maintain Gut and Systemic Immune Homeostasis. Cells.

[43] Rosser E.C., Piper C.J.M., Matei D.E., Blair P.A., Rendeiro A.F., Orford M., Alber D.G., Krausgruber T., Catalan D., Klein N. (2020). Microbiota-Derived Metabolites Suppress Arthritis by Amplifying Aryl-Hydrocarbon Receptor Activation in Regulatory B Cells. Cell Metab..

[44] Wirthgen E., Hoeflich A., Rebl A., Günther J. (2018). Kynurenic Acid: The Janus-Faced Role of an Immunomodulatory Tryptophan Metabolite and Its Link to Pathological Conditions. Front. Immunol..

[45] Mezrich J.D., Fechner J.H., Zhang X., Johnson B.P., Burlingham W.J., Bradfield C.A. (2010). An Interaction between Kynurenine and the Aryl Hydrocarbon Receptor Can Generate Regulatory T Cells. J. Immunol..

