# Supplementary material for: IL-33 Induces a Switch in Intestinal Metabolites Revealing the Tryptophan Pathway as a Target for Inducing Allograft Survival
Source: Nutrients. 2024 Oct 27;16(21):3655. doi: 10.3390/nu16213655 (PMC11547499; doi:10.3390/nu16213655)
Supplement: Supplementary file 1 [file nutrients-16-03655-s001.zip › nutrients-3270842-supplementary.pdf]

Supplementary Materials

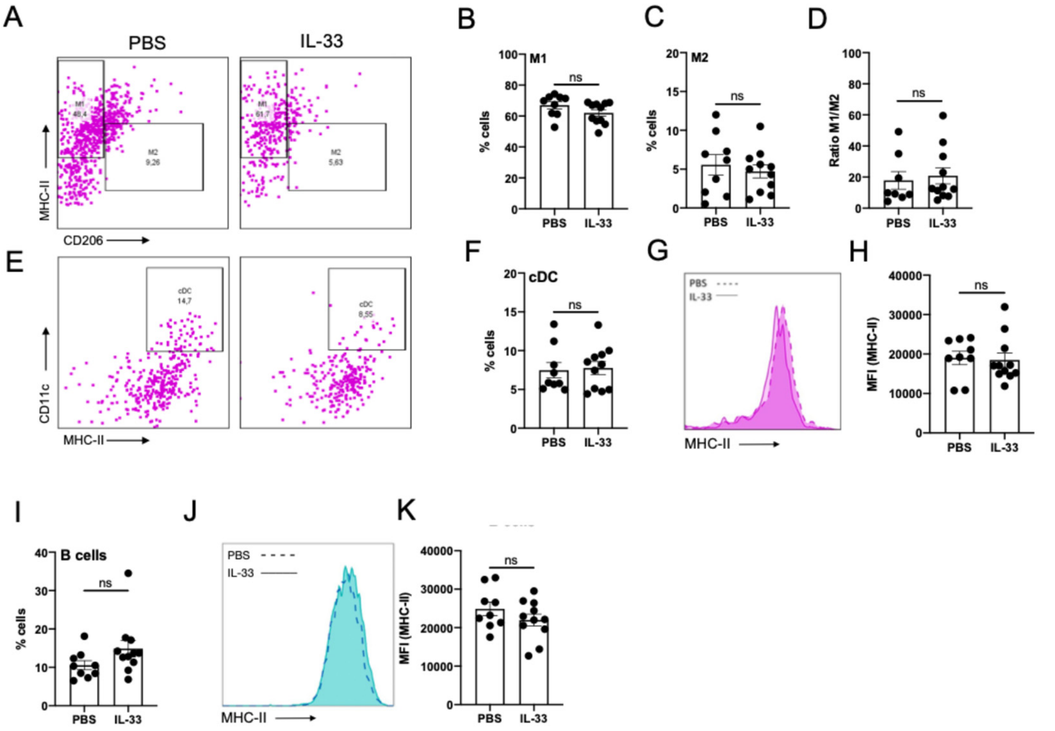

Figure S1. Effect of IL-33 on antigen-presenting cells. Mice were treated as mentioned in Figure 1. On day 4, MLNs were removed, and cell suspensions were incubated with antibodies to identify M1, M2, DC, and B cells. (**A**). Representative zebra plot to distinguish type 1 (M1:CD45+CD11b+MHC-IIint/high CD206-) and type 2 (M2:CD45+CD11b+MHC-IIint/low CD206+) macrophages. Graphs showing the frequencies of M1 (**B**) and M2 (**C**) and the M1/M2 ratio (**D**). (**E**). Representative zebra plot to identify dendritic cells (DC: CD45+CD11b+CD11c+MHC-II+). (**F**). Graph showing cDC frequencies. (**G**). Representative histogram showing MHC-II expression on cDCs. (**H**). Graph showing the mean fluorescence intensity (MFI) for MHC-II on cDCs. (**I**). Graph showing the frequencies of B cells (CD45+CD11b-CD19+). (**J**). Representative histogram showing MHC-II expression on B cells. (**K**). Graph showing the mean fluorescence intensity (MFI) for MHC-II on B cells. Each experiment had n = 3–5 mice per group. *** $p < 0.001$ according to the Mann–Whitney test; ns, not significant. Bar lines represent mean ± SEM.

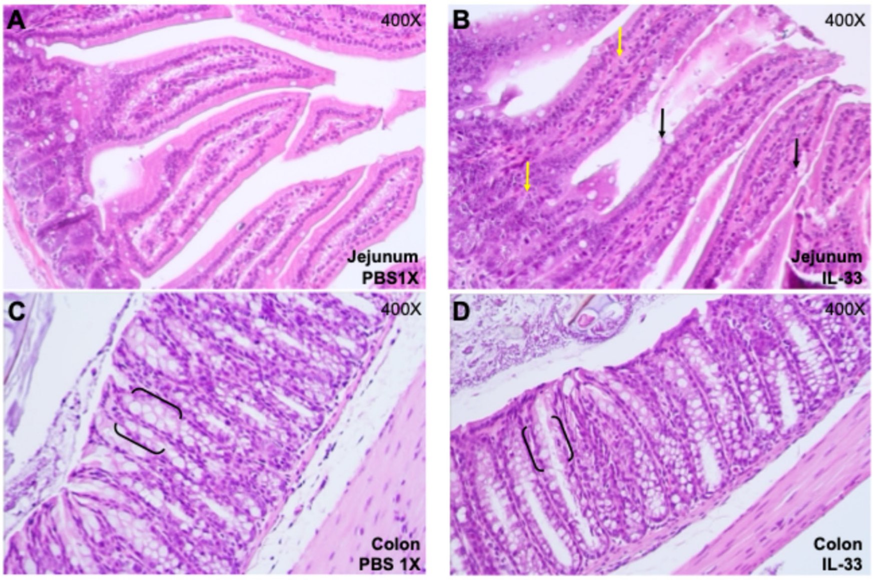

Figure S2. Intestinal histological analysis of IL-33-treated mice. C57BL/6 mice were treated with PBS 1X or 500 ng of IL-33 via i.p injection for 4 days. The intestine was removed, cleaned, and cut to collect the sections

corresponding to the jejunum and colon. Tissues were subjected to hematoxylin–eosin staining, as described in the Material and Methods section. Jejunum section of control (**A**) and IL33-treated animals (**B**). Colon sections of control (**C**) and IL-33-treated animals (**D**). Intraepithelial lymphocytes, neutrophils, and goblet cells are indicated with black and yellow arrows and brackets, respectively. Representative images of two independent experiments.

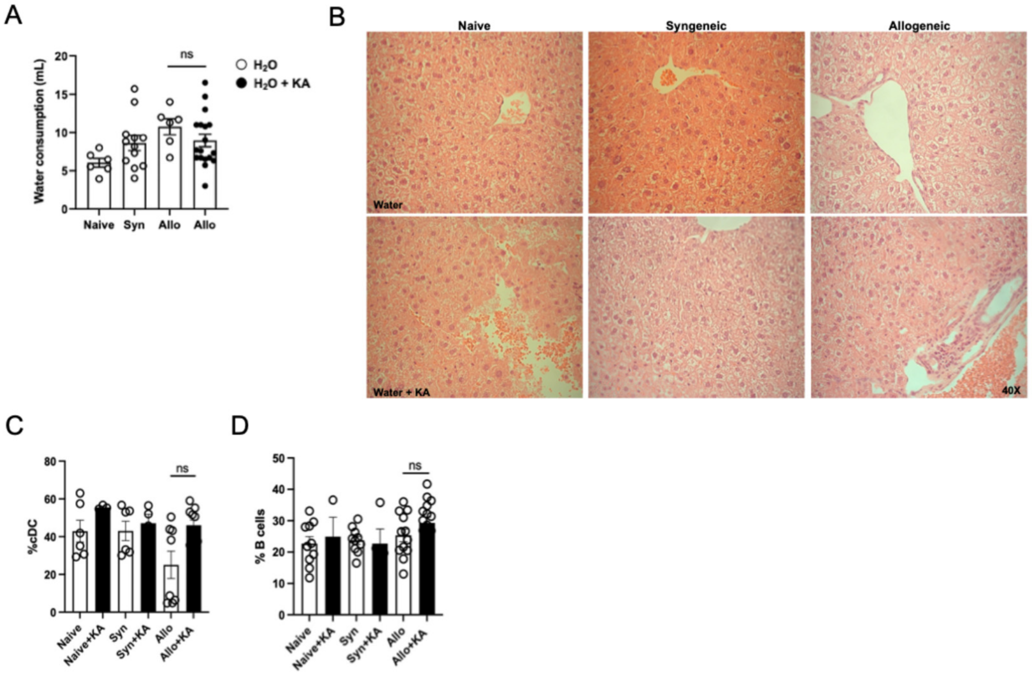

Figure S3. Effect of KA on skin transplant animals. Mice were transplanted and treated with KA as in Figure 5. (**A**). Total water consumption per experimental group for the duration of the experiment (20 days). (**B**). On day 20, livers were removed and processed for H&E staining. (**C**,**D**). Frequencies of DCs (CD11c+MHC-II+) and B cells (CD19+) present in dLNs. Each experiment had n = 3–5 mice per group. The Mann–Whitney test was applied; ns, not significant. Bar lines represent mean ± SEM.
